# Incidence of HIV disclosure among HIV affected heterosexual partners using a community health worker led mechanism in rural Uganda; a quasi-experimental study

**DOI:** 10.1186/s12879-023-08282-0

**Published:** 2023-05-11

**Authors:** Zubair Lukyamuzi, Bashir Ssuna, Ruth Nabisere Mirembe, Denis Mawanda, Joel Maena, Rita Nakalega, Patience Atuhaire, Philippa Musoke, Lisa M. Butler

**Affiliations:** 1 Makerere University, Johns Hopkins University (MU-JHU) Research Collaboration, Upper Mulago Hill Road, Kampala, Uganda; 2 Makerere University College of Health Sciences, School of Public Health, Kampala, Uganda; 3 Uganda Tuberculosis Implementation Research Consortium (U-TIRC), Kampala, Uganda; 4 Infectious Diseases Institute (IDI), College of Health Sciences, Makerere University, Kampala, Uganda; 5 Queen’s University, Kingston, ON Canada

**Keywords:** Incidence of disclosure, Community health worker, Adults living with HIV, Heterosexual relationships, Rural settings

## Abstract

**Background:**

HIV disclosure is vital in HIV management. Community Health Workers (CHW) were reported to support partner disclosure among HIV affected heterosexual partners with disclosure difficulties. However, time to disclosure attributed to use of CHW led disclosure support mechanism was not documented. This study compared the incidence of sexual partner disclosure among adults living with HIV (ALHIV) with CHW support and those without in the greater Luwero region, Uganda.

**Methods:**

We conducted a quasi-experimental study with two arms allocated by geographically determined clusters and adjusted for between-group differences; among ALHIV in the greater Luwero region of Uganda who had never disclosed to their current primary sexual partners. We allocated study clusters to either a CHW-led intervention or control arm. In both arms, we consecutively recruited participants; those in the intervention arm received CHW disclosure support in addition to routine care. The overall follow-up was six months, and the primary outcome was disclosure to the partner. We used survival analysis with proportional hazard ratios to determine the time to partner disclosure in both arms.

**Results:**

A total of 245 participants were enrolled, and 230 (93.9%) completed the study; of these, 112 (48.7%) were in the intervention and 118 (51.3%) in the control arm. The mean age was 31 ± 8 years with a range of 18 to 55 years; the majority were females, 176 (76.5%). The cumulative incidence of disclosure was higher in the intervention arm, 8.76 [95% CI: 7.20–10.67] per 1,000 person-days versus 5.15 [95%CI: 4.85–6.48] per 1,000 person-days in the control arm, log-rank test, X2 = 12.93, *P* < 0.001.

Male gender, aHR = 1.82, tertiary education, aHR = 1.51, and relationship duration of > six months, aHR = 1.19 predicted disclosure. Prior disclosure to a relative, aHR = 0.55, and having more than one sexual partner in the past three months, aHR = 0.74, predicted non-disclosure.

**Conclusion:**

CHW-led support mechanism increased the rate of sexual partner disclosure among ALHIV with disclosure difficulties. Therefore, to achieve the global targets of ending HIV, near location CHW-led disclosure support mechanism may be used to hasten HIV disclosure in rural settings.

## Introduction

Human Immunodeficiency Virus (HIV) remains a major public health problem worldwide with 38 million people globally infected by 2020 [1]. Despite the global commitment to reduce AIDS-related deaths and new HIV infections to fewer than 500 000, 650 000 AIDS-related deaths and 1.5 million new infections were registered in 2021 [2]. Although Sub-Saharan Africa (SSA) contributes only 12.0% of the world’s population, the region remains an epicenter for HIV/AIDS with 71.0% of the world’s HIV infections; with Eastern and Southern Africa being the most affected regions [1, 3]. Uganda remains one of the most affected countries with 1.4 million people living with HIV (PLHIV), 54,000 new infections, and 17,000 HIV/AIDs related deaths in 2021 [4].

Although HIV affects all age groups, adults are the most affected, especially those in sexual partnerships [3, 5, 6]; in Uganda, approximately 10.0% of sexual couples are affected by HIV [3], and a significant number of these couples have not disclosed individual HIV status to each other [7–9]. In the management of HIV, HIV status disclosure is fundamentally important [10–12], but non-disclosure remains common, and it is a critical challenge that hinders desirable HIV treatment outcomes [13–15]. Moreover, non-disclosure of HIV status among PLHIV in sexual relationships is associated with poor ART adherence, development of resistant strains, and increased HIV transmission [16–18]. In contrast, disclosure improves prevention and care outcomes in HIV management [19, 20], promotes social support and a sense of well-being, and enhances trust and social acceptance [21–23]. Despite its documented benefits, disclosure is always hindered by anticipation of negative outcomes, such as couple separation among those intending to disclose [24–26].

Disclosure among sexual partners is a process [25] that is influenced by various factors [27, 28] which can be categorized into barriers and facilitators depending on a specific sexual relationship [29–31]. For instance, disclosure is generally lower among casual sexual partners compared to regular partners. Similarly, financial dependence, literacy, having many sexual partners, not being on ART, lack of communication skills, younger age, and female gender decrease the chances of disclosure [8, 9, 20, 22]. On the other hand, receipt of regular disclosure counseling, longer duration in HIV care, being a member of an HIV/AIDS association, having the responsibility to disclose, and peer support increase the chances of disclosure [8, 9, 19, 29]. Additionally, the time taken to disclosure varies widely from the time of HIV diagnosis to many years of living with the disease [19]. Moreover, the shorter the time to disclosure among PLHIV, the quicker to achieve the desirable HIV management outcomes [32, 33].

Disclosure can be done by the PLHIV themselves or by significant others such as health workers on behalf of the person living with HIV after their consent [29]. Professional-facilitated couple counseling and assisted partner notification (APN) may quicken disclosure. However, trained health professionals are scarce in low-resource settings and such approaches are more facility-based than community-based [34–37]. Community Health Workers (CHW) are members of the communities where they come from and have basic training in providing various basic healthcare services, including home visiting, health promotion, and education, mobilization for immunization services, and supporting HIV care services such as linkage to care, HIV counseling, and ART adherence support [38–43]. CHW also support disclosure among ALHIV in heterosexual relationships [44]. However, the time to disclosure attributed to the use of the CHW-led disclosure support mechanism was not documented. Therefore, this study aimed to compare time to disclosure among ALHIV in heterosexual relationships with CHW support and those without in the greater Luwero region.

## Methods

The methods of this study were primarily used to compare the proportion of partner disclosure among ALHIV with CHW support and those without [44].

### Study design

**Fig. 1:**
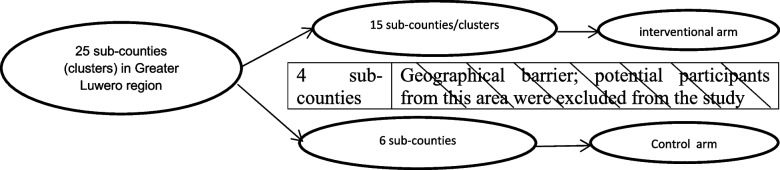
Recruitment of participants from their respective clusters

We conducted a quasi-experimental study with two study arms, and the allocation units were geographically demarcated clusters (sub-counties) of the greater Luwero region in Uganda. From October 3rd, 2019 to May 31st, 2020, we consecutively recruited ALHIV in sexual relationships who had never disclosed to their primary sexual partners. Participants from geographically adjoining clusters were allocated either an intervention (CHW support) or control arm (without CHW support) and followed up for six months. We compared the time to disclosure between the study arms at the end of the follow-up. Because there was a need for interaction between participants and CHW within the community for those allocated to the intervention arm, we utilized some clusters as a geographical barrier (buffer zone) between the intervention and control clusters. All potential participants from the buffer zone were excluded from the study as shown in Fig. 1 [44].

### Study area and population

The study area was the greater Luwero region, located about 50 km from Kampala, the capital of Uganda, and comprised of Luwero, Nakaseke, and Nakasongola districts. The three region districts had a total of about 2,000 CHW [42]. The region also comprised 25 sub-counties, which served as clusters in this study. In Uganda, a sub-county was made up of about six parishes (which were made up of several villages or zones) and was run by a technical sub-county chief and elected local council III chairman. Of the 25 sub-counties, 15 adjoining sub-counties were allocated to the intervention arm, six to the control, and four formed the buffer zone. Because of the prior administratively determined geographical sizes, sub-counties in the control arm were relatively bigger, and thus fewer than those in the intervention. The region had a population of about 949,100 [45], served by four hospitals, and seven health center IVs (HCIV). In Uganda, a hospital provided all surgical and medical services while an HCIV provided general health services plus minor surgeries [46, 47]. Therefore, the study sites were high volume (busiest) HIV care units in Luwero, Nakaseke, and Kiwoko hospitals; Semuto, Nyimbwa, Kalagala, Ngoma, Nakasongola, Nabiswera, and St. Francis HCIV. We were not able to work in the fourth hospital because it granted us administrative approval towards the end of the study.

Participants in the study were ALHIV who had been in heterosexual relationships for at least three months and had not disclosed their status to their current primary partner. Participants enrolled in study arms in which their respective sub-counties had been allocated.

### Community health worker intervention

As previously described in the primary study [44], the intervention was the CHW-led disclosure support mechanism which is described as follows; after enrollment, each participant in the intervention arm was asked to provide the name and contact information (if they were known to the participant) of a CHW in their area of residence and the obtained details were verified in the CHW district registry. The verified CHW were contacted and informed about the study, and were scheduled for training. A total of 48 CHW aged between 25 and 60 were recruited and trained for three days about HIV basic counseling skills, HIV status disclosure skills, health ethics, confidentiality and privacy, and management of adverse events associated with disclosure. Trainings were done using both role play and didactic model, moderated by HIV care counselors and study investigators. Pre and post training assessments were done.

In addition to the routine care, participants in the intervention arm were linked and attached to trained CHW from their respective areas of residence. One hundred and twenty-one participants were paired with 48 CHW irrespective of gender in the ratio of 3:1. After pairing the participant and the CHW initially met and laid out a specific disclosure plan. The plan generally included two weekly phone calls and scheduled home visits. Discussions during phone calls and home visits included methods or skills to be used in the disclosure, assessment of the partner’s attitude toward HIV and personality, potential adverse outcomes and how to overcome them, and the partner’s availability and timings at home. They also practiced how to start and handle the disclosure process. Depending on the agreements from the above discussions, eventual disclosure would occur at the home of the participant or the health facility, according to the participant’s preference and choice as described previously [44].

At the participant’s home, disclosure to their partner would be done in the presence or absence of a CHW, or in the presence of the participant, the CHW would disclose to the participant’s partner on the participant’s behalf. Otherwise, the CHW would encourage and arrange for couple HIV testing and counseling at the participant’s preferred health facility where the facility counselor would further support disclosure.

CHW received ongoing supervision via regular phone calls and meetings with the study team. They completed home visit and phone call logs whenever they visited or telephoned the participant for study purposes, and they received a monthly facilitation and transport allowance of 50,000 Ugx ($15).

### Control (routine care)

Participants in the control arm continued to receive standard of care at their respective HIV care centers (study sites). This involved HIV counseling and disclosure counseling at every routine care appointment visit, anti-retroviral refills, adherence counseling, and psychosocial support. With routine care, participants would disclose by themselves at their homes or persuade their partners to go to the health facility for couple HIV counseling and testing where a facility counselor would support disclosure.

### Outcomes

The primary outcome was time to disclosure during the six months’ follow-up. For participants in the intervention arm, information about the occurrence of disclosure was obtained from the participants and verified from CHW. For the control arm, we collected disclosure information from only participants. We collected disclosure information at three monthly subsequent in-clinic study visits. Disclosure information was verified with CHW, and confirmed from the non-study partners of those participants who permitted the study team to contact their partners. Otherwise, all participants were encouraged to bring their partners to the study site or health facility for HIV counseling and testing.

### Independent variables were

CHW intervention, age, education, gender, marital status, nature of marriage, duration of the relationship, monthly income, partner HIV status, duration on ART, condom use, person previously disclosed to, membership to an HIV/AIDs association, place of HIV diagnosis, prior receipt of disclosure counseling, negative attitude towards other people knowing one’s HIV status, feeling of responsibility to disclose, and ever had chance(s) to disclose (such as partner asking for couple HIV testing).

### Sample size and sampling procedures

We estimated the sample size using the Fleiss formula for two proportions, [48];

$N=\frac{\left[z_{\alpha}\sqrt{P\left(1-P\right)\left(1/q_{1}+1/q_{2}\right)}+z_{\beta}\sqrt{P_{1}\left(1-P_{1}\right)\left(1/q_{1}\right)+P_{2}\left(1-P_{2}\right)\left(1/q_{2}\right)}\right]^2}{\left(P_{1}-P_{2}\right)^2}$, where p1 was assumed to be the proportion of control arm participants expected to disclose at the end of the study, p2 was the assumed proportion of intervention arm disclosure, q1 was the assumed proportion of non-disclosure in the control arm, and q2 was the assumed proportion of non-disclosure in the intervention arm. P = q1p1 + q2p2, and N was the total number of participants. We used a baseline disclosure of 54.0% [8] and assumed a disclosure increase of 46.0% by the intervention based on the previous study in which CHW improved tuberculosis sputum positive case detection [49]. Therefore, using the information above, we got the expected proportion of disclosure in the intervention group to be 0.78. We set power at 80.0% (Zβ = 0.84), design effect of 2, and alpha at 0.05 (Zα = 1.96). Thus, the total estimated sample size was approximately 236.

### Data collection procedures

As mentioned earlier, the data collection procedures of the current study followed the primary study [44]; for both study arms, we recruited participants between 3rd October 2019 and 7th November 2019. All ALHIV who came in for HIV services at the study sites during the above period were informed about the study; those who were interested were consented and consecutively enrolled. We enrolled about eight participants per day across all sites. The eligibility criteria were: an adult (above 18 years), HIV positive irrespective of the ART status, being in a heterosexual relationship for at least three months, having not disclosed their HIV status to their current primary sexual partner, having stayed in the study area for at least three months, and willing to provide informed consent. Potential participants from the buffer zone were excluded from the study to minimize contamination. All participants completed a questionnaire at enrollment and a disclosure assessment form at three and six-month in-clinic visits. Participants in different study arms who attended the same study site were enrolled on the different dates and given different appointment dates for subsequent in-clinic visits to minimize cross contamination at the study site. All participants continued with routine HIV care. However, those in the intervention arm received CHW disclosure support in addition.

The study's endpoints (events) were HIV disclosure during the six-month follow-up. Participants who experienced adverse events such as fighting and separation during the study period were kept in the study until the end of the follow-up as they continued to receive reconciliation counseling (dispute resolution in case the partner was reachable) and social support from the study team.

### Statistical analysis

Data were collected using REDcap_v8.5.11, then transferred into an excel sheet and later to STATA 15/MP for analysis [50]. Univariately, data were summarized using descriptive texts, tables, and graphs. We summarized continuous variables like age as mean with standard deviation (SD) and median with interquartile range. We summarized categorical variables as frequencies and proportions. We used a Chi-square to test for the baseline difference between frequencies of different variables. We computed survival functions using Kaplan Meier; and we presented survival curves. We determined survival as the number of participants who failed to disclose, and we calculated it by following participant’s exact survival times. We calculated disclosure rate as the number of participants who disclosed their HIV status divided by the total number of participants recruited expressed as number per 1,000 person-days. The probability of the event (disclosure) was the number of events at that time divided by the number at risk at that point in time. Cox regression analysis was used to assess for the predictors of disclosure or non-disclosure at the end of follow-up and presented as Hazard ratios (HR) with their 95.0% confidence intervals (CI) at both bivariate and multivariable levels. All variables with a *P* < 0.2 and those known to influence disclosure, such as membership to an HIV/AIDs association, financial dependence, literacy, prior receipt of disclosure counselling, number of sexual partners, and ART status were entered in the multivariable cox regression. A *p*-value of < 0.05 was considered statistically significant. We assessed for interactions and confounding, and all variables that cause a ≥ 10.0% change in crude and adjusted models were considered confounders.

## Results

**Fig. 2:**
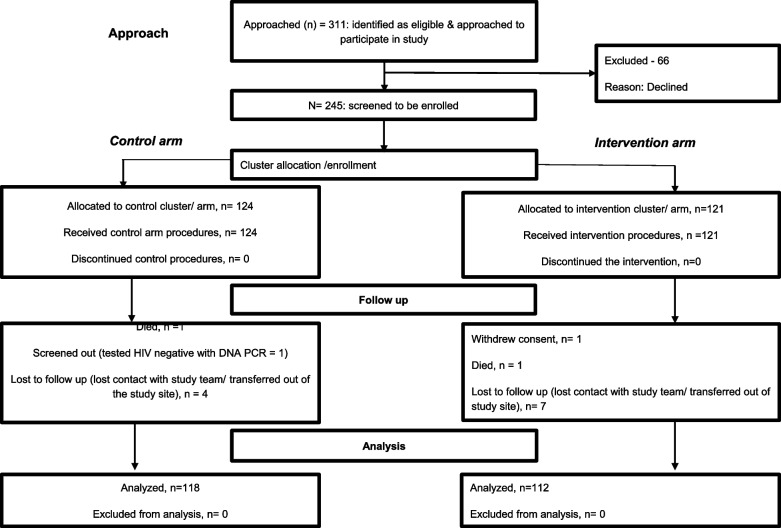
The number of participants evaluated at each stage of the study in rural Uganda

Screening and enrollment procedures were done at the study sites and participants were recruited and allocated study arms as shown in the Fig. 2 [44].

### Description of study participants

A total of 245 participants were recruited from 10 health facilities with an average of 25 participants per facility. Two facilities recruited participants in both study arms, and the rest recruited in either study arm. A total of 230 (93.9%) participants completed the study and of these, 112 (48.7%) were in the CHW intervention while 118(51.3%) were in the control arm. Luwero and Kiwoko hospitals enrolled participants in both arms. Overall, Luwero hospital enrolled 48 participants (36 in the intervention arm and 12 in control), and Kiwoko hospital enrolled 33 participants (26 in the intervention arm and 7 in control). The rest of the other facilities enrolled 20 participants on average in either the intervention or control arm as previously reported [44]. The median age was 30(IQR = 25–37) years. The majority of those recruited in the CHW intervention arm were females, 95 (84.8%). Between the study arms, there were significant differences between gender, *p-value* = *0.004*, education status, *p-value* = *0.019*, marital status, *p-value* = *0.008*, nature of marriage, *p-value* = *0.019*, and need for healthcare worker disclosure support, *p-value* < *0.001* as shown in Table 1.

**Table 1:** Demographic characteristics of participants in the intervention compared to control arm

Characteristic	Sample size (%) *N* = 230	Intervention n (%) *N* = 112	Control n (%) *N* = 118	*P*-value
**Age group (years)**				0.102
18–35	163(70.9)	85(75.9)	78(66.1)	
35–55	67(29.1)	27(24.1)	40(33.9)	
**Gender**				0.004
Female	176(76.5)	95(84.8)	81(68.6)	
Male	54(23.5)	17(15.2)	37(31.4)	
**Education**				0.019
None	30(13.0)	12(10.7)	18(15.3))	
Primary	122(53.1)	52(46.4)	70(59.3)	
Secondary	67(29.1)	39(34.8)	28(23.7)	
Tertiary	11(4.8)	9(8.0)	2(1.7)	
**Marital status**				0.008
Casual partner	60(26.1)	22(19.6)	38(32.2)	
Cohabiting	139(60.4)	68(60.7)	71(60.2)	
Married	21(9.1)	22(19.6)	9(7.6)	
**Nature of Marriage**				0.019
Monogamous	13(5.7)	8(7.1)	5(4.2)	
Polygamous	18(7.8)	14(12.5)	4(3.4)	
Not mentioned	199((86.5)	90(80.4)	109(92.4)	
**Duration of relationship**				0.521
< 6 months	14(6.1)	5(4.5)	9(7.6)	
6 months – 1 year	57(24.8)	30(26.8)	27(22.9)	
> 1 year	159(69.1)	77(68.8)	82(69.5)	
**Monthly income**				0.989
< 120,000	147(63.9)	72(64.3)	75(63.6)	
120,000 – 500,000	77(33.5)	37(33.0)	40(33.9)	
> 500,000	6(2.6)	3(2.7)	3(2.5)	
Partner’s circumcision status				0.371
No	79 (44.6)	39 (49.4)	40 (50.6)	
Yes	97 (55.4)	54 (56.1)	43 (43.9)	
**Reported a need of Health work disclosure support**				< 0.001
No	93 (40.4)	18 (19.4)	75 (80.6)	
Yes	137 (59.6)	94 (68.6)	43 (31.4)	

### Behavioural and HIV associated characteristics

The majority 184 (80%) of the participants didn’t know their sexual partner’s HIV status. Among sexual partners whose HIV status was known by participants, 38/40 (82.6%) were negative. All participants were on ART and 194 (79.8%) had disclosed to either a friend or relative and 51 (20.8%) had never disclosed to anyone. Thirteen (5.7%) participants were members of the HIV group or association (such as peer educators). The majority, 182 (79.1%) participants had received prior partner disclosure counselling by a health care worker. The majority of the participants 120 (52.2%) reported having a negative attitude towards other people knowing their HIV status. Between the study arms, there were significant differences in knowing the partner's HIV status, *p*-value = 0.049, duration on ART, *p*-value = 0.001, place of HIV testing, *p*-value = 0.033, prior receipt of disclosure counselling, *p*-value = 0.004, having a negative attitude towards other people knowing one’s HIV status, *p*-value = 0.005, and previously had a conducive disclosure environment, *p*-value =  < 0.001 as shown in Table 2.

**Table 2:** Clinical and behavioural characteristics of participants in the intervention compared to control arm

Characteristic	Sample size (%) *N* = 230	Intervention n (%) *N* = 112	Control n (%) *N* = 118	*P*-value
**Partner HIV status**				0.049
Negative	38(16.5)	12(10.7)	26(22.0)	
Positive	8(3.5)	3(2.7)	5(4.2)	
Don’t know	184(80.0)	97(86.6)	87(73.7)	
**Duration on ART**				0.001
< 6 months	55(23.9)	38(33.9)	17(14.4)	
6 months- 1 year	31(13.5)	10(8.9)	21(17.8)	
> 1 year	144(62.6)	64(57.1)	80(67.8)	
**Condom use**				0.191
No	130(56.5)	68(60.7)	62(52.5)	
Sometimes	83(36.1)	39(34.8)	44(37.3)	
Always	17(7.4)	5(4.5)	12(10.2)	
**Person disclosed to**				0.050
No	39(17.0)	24(21.4)	15(12.3)	
Friend	21(9.1)	6(5.4)	15(12.7)	
Relative	170(73.9)	82(73.2)	88(74.6)	
**Place of HIV testing**				0.033
ANC Clinic	59(25.7)	32(28.6)	27(22.9)	
VCT Clinic	149(64.8)	75(67.0)	74(62.7)	
Other	22(9.6)	5(4.5)	17(14.4)	
**Membership to HIV/AIDS association**				0.127
No	217(94.3)	103(92.0)	114(96.6)	
Yes	13(5.7)	9(8.0)	4(3.4)	
**Prior receipt of disclosure counselling**				0.004
No	48(20.9)	27(24.1)	21(17.8)	
Always	37(16.1)	8(7.1)	29(24.6)	
Only at testing	75(32.6)	42(37.5)	33(28.0)	
Sometimes	70(30.4)	35(31.3)	35(29.7)	
**Negative attitude towards other people knowing one’s HIV status**				0.005
No	110(47.8)	43(38.4)	67(56.8)	
Yes	120(52.2)	69(61.6)	51(43.2)	
**Feeling a responsibility to disclose**				0.140
No	22(9.6)	14(12.5)	8(6.8)	
Yes	208(90.4)	98(87.5)	110(93.2)	
**Having a conducive disclosure environment**				
No	166(72.2)	93(83.0)	73(61.9)	< 0.001
Yes	64(27.8)	19(17.0)	45(38.0)	
**Number of sexual partners in the last 3 months**				
None	4 (1.7)	2 (50.0)	2 (50.0)	0.700
One	60 (26.1)	32 (53.3)	28 (46.7)	
More than one	166 (72.2)	78 (47.0)	88 (53.0)	

### Time to disclosure to sexual partner among ALHIV

**Fig. 3:**
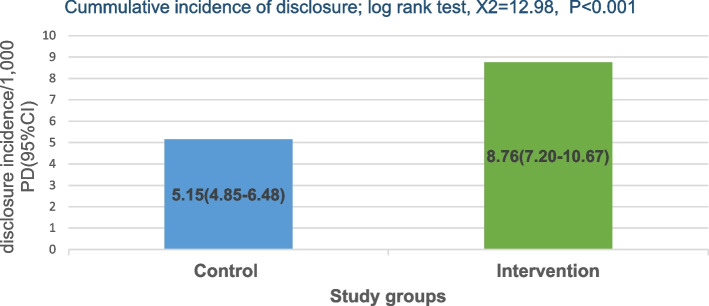
Cumulative incidence of disclosure among the study groups

**Fig. 4:**
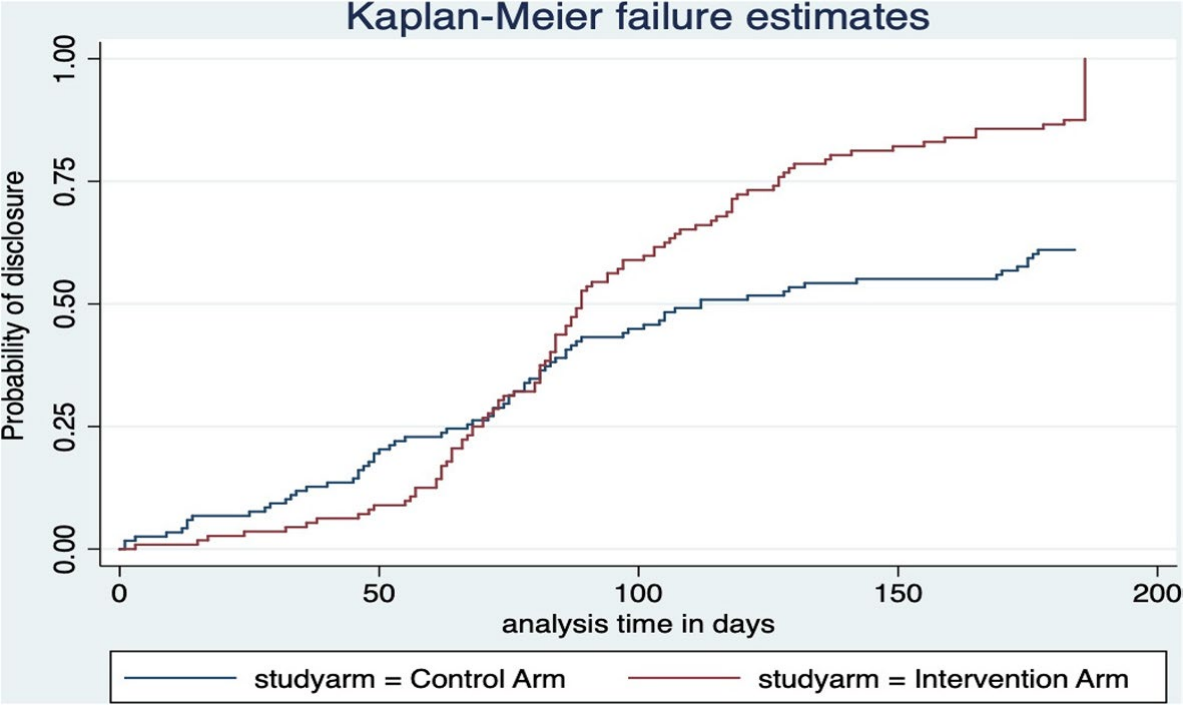
Kaplan Meier failure curve showing disclosure to sexual partners among ALHIV by study arm

Two hundred thirty (230) participants were followed up for a total period of 25,290 person-days. One hundred and eighteen (51.3%) participants were in the control arm with a total follow-up period of 13,992 person-days while 112 (48.7%) were in the intervention arm with a total follow-up period of 11,298 person-days. The median time of disclosure was 82 days with an interquartile range (IQR) of 61–107 days; with the median time to disclosure of 89 days (IQR:69–127) in the intervention arm and 112 days (IQR: 67–184) in the control arm. In the control arm, 72/118 (61%) participants disclosed their HIV status within the follow-up period at a disclosure rate of 5.15 [95%CI: 4.85–6.48] per 1,000 person-days while 99/112 (88.4%) participants disclosed in the intervention arm within the follow-up period at a rate of 8.76 [95% CI: 7.20–10.67] per 1,000 person-days. The probability of disclosure between the control and intervention arms was statistically significant by log-rank test, X2 = 12.93, *P* < 0.001 as shown in Fig. 3. In the first 50 days of follow-up, the probability of disclosure was slightly higher in the control arm than in the intervention arm. However, the probability of disclosure exponentially increased in the subsequent 70 days for the intervention arm nearly doubling that in the control arm as shown in Fig. 4.

Among the partners who were disclosed to, 104/171 (60.8%) accepted to come to the study site for HIV counselling and testing; of these, 55 (52.9%) tested negative, 23 (22.1%) confessed to being HIV positive and already in HIV care, and 26 (25%) newly tested positive (were linked to HIV care).

### Predictors of HIV status disclosure to sexual partners among ALHIV

Male gender, aHR = 1.82 [95% CI: 1.26–2.65], tertiary education, aHR = 1.51 [95% CI: 1.43–1.60], and relationship duration of > 6 months, aHR = 1.19 [95% CI: 1.16–1.22] predicted disclosure. Prior disclosure to a relative, aHR = 0.55 [95% CI: 0.39–0.78] and having more than one sexual partner in the past three months, aHR = 0.74 [95% CI: 0.60–0.92] predicted non-disclosure as shown in Table 3.

**Table 3:** Predictors of disclosure to sexual partners among ALHIV

Characteristic	Frequency (%)	Crude HR (95% CI)	Adjusted HR (95% CI)	*P*-value
**Gender**				
Female	176(76.5)	1.00	1.00	
Male	54(23.5)	1.57 [1.12–2.20]	**1.82 [1.26–2.65]**	**0.002**
**Education**				
None	30(13.0)	1.00	1.00	
Primary	122(53.1)	1.08 [0.66–1.76]	1.22 [0.42–3.53]	0.709
Secondary	67(29.1)	1.18 [0.71–1.98]	1.29 [0.51–3.27]	0.590
Tertiary	11(4.8)	1.86 [0.87–3.97]	**1.51 [1.43–1.60]**	**< 0.001**
**Duration of relationship**				
< 6 months	14(6.1)	1.00	1.00	
6 months – 1 year	57(24.8)	0.61 [0.47–0.80]	**1.19 [1.16–1.22]**	**< 0.001**
> 1 year	159(69.1)	0.61 [0.57–0.66]	**1.32 [1.32–1.33]**	**< 0.001**
**Prior person disclosed to**				
None	39(17.0)	1.00	1.00	
Friend	21(9.1)	0.35 [0.34–0.36]	0.35 [0.04–2.95]	0.336
Relative	170(73.9)	0.56 [0.40 -0.78]	**0.55 [0.39–0.78]**	**0.001**
**Place of HIV testing**				
ANC Clinic	59(25.7)	1.00	1.00	
VCT Clinic	149(64.8)	1.12 [1.01–1.24]	**1.36 [1.34–1.39]**	**< 0.001**
Other	22(9.6)	1.24 [0.66–2.33]	1.76 [0.62–5.00]	0.290
**Prior receipt of disclosure counselling**				
No	48(20.9)	1.00	1.00	
Always	37(16.1)	0.54 [0.45–0.66]	0.84 [0.57–1.25]	0.400
Only at testing	75(32.6)	1.33 [1.10–1.62]	2.13 [0.69–6.62]	0.191
Sometimes	70(30.4)	0.50 [0.45–0.56]	**0.61 [0.38–0.98]**	**0.040**
**Reported a need of Health care worker disclosure support**				
No	93 (40.4)	1.00	1.00	
Yes	137 (59.6)	1.44 [0.93–2.22]	1.66 [0.90–3.05]	0.105
**Number of sexual partners in the last 3 months**				
None	4 (1.7)	1.00	1.00	
One	60 (26.1)	0.26 [0.05–1.32]	0.68 [0.39–1.21]	0.189
More than one	166 (72.2)	0.39 [0.10–1.49]	**0.74 [0.60–0.92]**	**0.006**
**Partner circumcision status**				
No	79 (44.6)	1.00	1.00	
Yes	97 (55.4)	1.51 [1.22–1.85]	**1.32 [1.13–1.13–1.53]**	** < 0.001**

## Discussion

In this study, we aimed to determine the incidence of disclosure among ALHIV in heterosexual relationships attributed to the use of CHW-led disclosure mechanism compared to routine care; and we found out that CHW increased the incidence of HIV disclosure from 5.15 per 1,000 person-days to 8.76 per 1,000-person-days. Male gender, tertiary education, index HIV testing at the VCT clinic, having a circumcised partner, and relationship duration of > six months predicted disclosure. However, prior disclosure to a relative and having > 1 sexual partner in the past three months predicted non-disclosure.

The findings suggest that the CHW-led disclosure mechanism quickened disclosure in rural Ugandan settings among HIV-affected sexual partners. To our knowledge, this is the first study to determine the rate of sexual partner disclosure among ALHIV using a CHW-led mechanism. This means that near location disclosure support mechanism that comprises home visiting, phone calls, and skill building by CHW is faster in achieving disclosure among ALHIV with difficulties when compared to routine care. This finding was similar to previous reports where CHW increased the proportion of disclosure among ALHIV with partner disclosure difficulties [51]. Similarly, Exavery et al. reported that community-based interventions accelerated HIV disclosure among caregivers of Orphans and Vulnerable Children to community-based lay social welfare volunteers [52]. Therefore, the results of the current study emphasize the fundamental role of CHW in improving HIV care and reducing the workload of Healthcare workers which was previously reported [53–57]. However, some previous reports have not shown the significant importance of CHW-based interventions in HIV management [58–60]. This difference from the current study could have been due to variations in study designs, populations, and settings. The current findings may propose that as the world is struggling to achieve the nearing global targets of ending HIV/AIDs by 2030 [32], using effective and faster CHW-led HIV disclosure support mechanism may be critically important.

In this study, the male gender predicted disclosure, a similar finding reported in previous reports [19, 51, 61–63]. Men’s HIV disclosure in the current study could be due to financial independence, and reduced fear of financial support implications following disclosure which is common in women [29–31]. Relatedly, men were reported to perceive less HIV-related stigma in a sexual relationship making them more likely to disclose than women [64]. However, some reports have shown that women are likely to disclose than men [65–67], and others have not found significant gender-specific differences [68]. These differences may probably be due to different study settings, interventions, and study populations.

Relationship duration of more than six months predicted disclosure. This implied that as the relationship lasts longer, there is more understanding of each other among the couple, trustworthiness, bonding, and stability compared to newer relationships. Moreover, new relationships are more prone to accusations of infidelity and promiscuity [69–71]. In Tanzania, disclosure was found to be directly proportional to the duration of the relationship [69]. Relatedly, in a study conducted by Mbichila et al., PLHIV in sexual relationships for more than one year had 0.82 more odds of partner disclosure when compared to those who had been in a relationship for less than one year [72].

Tertiary education predicted HIV disclosure which implied that a higher educational level may be associated with a better understanding of the benefits of disclosure or having an easier time when discussing personal, HIV, and intimate matters with a partner as previously reported [73]. Other studies also reported similar findings [71, 74–76]. Disclosure attributed to a higher level of education may also be explained by the fact that PLHIV with higher education levels are more comfortable and confident to disclose to their partners; and are more likely to be financially stable as opposed to those with lower levels of education [69]. Moreover, higher education level was reported to be protective against negative outcomes of disclosure such as gender-based violence [77]. Contrary to the current study, a study done in Tanzania did not find any association between educational level and HIV disclosure to a sexual partner [69]. This could have been due to differences in study populations. In the Tanzanian study, the study population was pregnant women as opposed to the ALHIV in the current study.

Prior disclosure to a relative predicted non-disclosure. Abdool et al. also found that initial disclosure to a relative was associated with 65% fewer chances of disclosure to a sexual partner [78]. A similar finding was also reported by Antelman et al. [69]. However, it is reported that the majority of PLHIV usually first disclose to their relatives to be emotionally supported and prepared or advised to disclose to significant others [79]. This provides psychosocial support and encourages the person living with HIV to disclose to the partner. These differences from the current study could be explained by the differences in the study populations and designs.

Having more than one sexual partner predicted also non-disclosure which was a similar finding in previous studies [20, 22, 69]. Having many sexual partners is socially judged as infidelity and promiscuous. Therefore, PLHIV with multiple sexual partners may not disclose or defer disclosure to avoid such judgments [9, 80, 81].

### Study strengths and limitations

To the best of our knowledge, this was the first study to empirically assess the relative incidence of partner disclosure among PLHIV with CHW support and those without. The creation of a buffer zone minimized intervention contamination among study arms due to the non-blinded nature of the study. The study depended not only on the participant's self-reported disclosure but also verified and confirmed disclosure with CHW and some non-study partners upon obtaining consent from the participant; this reduced social desirability bias.

The results of this study were limited by the non-randomized nature of the clusters, which may be prone to selection bias or confounding. Two study sites recruited participants in both study arms hence a possibility of intervention dilution; however, such sites first recruited participants in the intervention arm and then in the control arm. Moreover, participants of each study arm had different scheduled in-clinic visits hence minimizing the possibility of these participants meeting at the facility (study site). There was a variation in participants’ characteristics between study arms; this was probably due non-randomized nature of the study clusters and participants or disproportionate among PLHIV receiving HIV care in regards to gender (e.g., there were more women in HIV care than men). However, we adjusted for all predictor variables in the analysis.

There were no interactions during analysis. However, reported a need of health worker disclosure support was confounding with prior receipt of disclosure counselling but since prior receipt of disclosure counseling was significantly associated with disclosure, we retained the two confounding variables in the final analysis model.

## Conclusion

HIV disclosure in a sexual relationship improves HIV management outcomes. Therefore, to meet the global targets of ending HIV, CHW-led disclosure support mechanism may be used to quicken disclosure among ALHIV in sexual relationships with disclosure difficulties.

## Data Availability

The datasets used and/or analyzed during the current study are available from the corresponding author on reasonable request.
